# Alpha-lipoic acid supplementation restores the meiotic competency and fertilization capacity of porcine oocytes induced by arsenite

**DOI:** 10.3389/fcell.2022.943757

**Published:** 2022-10-03

**Authors:** Mianqun Zhang, Lei Sun, Zihao Zhang, Luyan Shentu, Yiwen Zhang, Ziyi Li, Yongteng Zhang, Yunhai Zhang

**Affiliations:** Anhui Province Key Laboratory of Local Livestock and Poultry Genetical Resource Conservation and Breeding, College of Animal Science and Technology, Anhui Agricultural University, Hefei, China

**Keywords:** alpha-lipoic acid, arsenite, meiotic defect, fertilization capacity, oocyte quality, oxidative stress, apoptosis

## Abstract

Arsenite is known as a well-known endocrine disrupting chemicals, and reported to be associated with an increased incidence of negative health effects, including reproductive disorders and dysfunction of the endocrine system. However, it still lacks of the research regarding the beneficial effects of ALA on arsenite exposed oocytes, and the underlying mechanisms have not been determined. Here, we report that supplementation of alpha-lipoic acid (ALA), a strong antioxidant naturally present in all cells of the humans, is able to restore the declined meiotic competency and fertilization capacity of porcine oocytes induced by arsenite. Notably, ALA recovers the defective nuclear and cytoplasmic maturation of porcine oocytes caused by arsenite exposure, including the impaired spindle formation and actin polymerization, the defective mitochondrion integrity and cortical granules distribution. Also, ALA recovers the compromised sperm binding ability to maintain the fertilization potential of arsenite-exposed oocytes. Importantly, ALA suppresses the oxidative stress by reducing the levels of ROS and inhibits the occurrence of DNA damage along with apoptosis. Above all, we provide a new perspective for the application of ALA in effectively preventing the declined oocyte quality induced by environmental EDCs.

## Introduction

As one of the most well-known Environmental Endocrine Disruptors (EDCs), arsenic is ubiquitous in the environment, arsenic in the drinking water, air, land, and living systems, is considered as one of the top environmental health threats in worldwide (Gao et al., 2021). Arsenic is found in the environment as oxides, with two different oxidation states: arsenite (As (III), as H_3_AsO_3_) and arsenate (As (V), as HAsO_4_^2-^). In the aqueous environment, the oxyanions consisting of arsenite species and arsenate species predominate. Arsenite species tend to predominate in groundwater under reducing conditions, whereas arsenate species are more frequently found under oxidizing conditions. The mobility of arsenic compounds in soils depends on the pH value, the redox potential, organic matter, clay and sand content, and other elements in the soil. It is noteworthy that arsenite is 100 times more toxic than arsenate because of its carcinogenic, teratogenic and mutagenic nature (Firrincieli et al., 2019). Since its high-affinity interaction with the ligand-binding domain of the estrogen receptors alpha, arsenite affects both the male and female reproductive systems. Humans are exposed to arsenite through a variety of ways, including groundwater consumption, diet and inhalation of airborne particulates matter from contaminated soil (Palma-Lara et al., 2020; Wu et al., 2020). Epidemiological data have revealed that arsenite can easily enter the bloodstream once ingested, and transported to the liver and distributed to body’s organs and tissues, eventually accumulated in the body (Bourguignon et al., 2017). Previous studies showed that prolonged exposure to arsenite cause peripheral vascular disease, increasing the incidence of lung, kidney, skin and liver cancers (Tryndyak et al., 2020). Arsenite also cause damage to the reproductive system in animals in different ways and to different degrees. For the male reproductive system, arsenite lowers the weight of testicular tissues and reduces the sperm counts along with the lower sperm motility, chromosomal aberrations and morphological sperm abnormalities (Wu et al., 2020). As for female, arsenite causes female masculinization in rats, ultimately impairing their normal reproductive function (Souza et al., 2020). In addition, arsenite delays the initiation of puberty in rats and increases the incidence of spontaneous abortion and stillbirth, accompanied with the presence of the antioxidant enzyme activity and sex hormone concentrations in the ovaries and uterus (Quansah et al., 2015). Female fertility is regulated by both the female reproductive system and the endocrine system (Yao et al., 2021). Thus, it is susceptible to the exposure of EDCs in the environment (Green et al., 2021). There is now overwhelming evidences that arsenite causes the accumulation of ROS and reactive nitrogen species (RNS) in somatic cell and male germ cells (Wang et al., 2013). ROS play important roles in the reproductive process including ovulation, nevertheless, excessive ROS and RNS attack important macromolecules and organelles inducing mitochondrial dysfunction, and thereby leading to the cell apoptosis (Guidarelli et al., 2017). For example, excess oxygen free radicals can damage oocytes at a genetic level through oxidative stress, causing infertility (Barati et al., 2020). However, the underlying mechanisms concerning how arsenite impacts the meiotic competence and fertilization capacity of porcine oocytes still remain unknown.

Alpha-lipoic acid (ALA), a natural care product in the health market, is a strong antioxidant present in all types of prokaryotic and eukaryotic cells (Di Nicuolo et al., 2021). ALA participates in aerobic metabolism, converting blood sugar (glucose) into energy consuming the oxygen (Erickson et al., 2020). In particular, ALA exerts the effects by scavenging free radicals, metal ion chelation and antioxidant recycling. Besides, ALA promotes the effectiveness of endogenous antioxidants such as glutathione and enhances their capacity to scavenge free radicals (Solmonson and DeBerardinis, 2018; Salehi et al., 2019). ALA has been identified to effectively scavenge ROS and eliminate the damage of the reproductive systems exposed to heavy metals, thereby ameliorating fertility in rats (Prathima et al., 2018).

In the current paper, porcine oocytes were used as the research model to study whether the application of ALA could prevent the declined oocyte quality induced by arsenite, because there are many similarities between porcine oocytes and human oocytes in aspects of physiological metabolism and pathogenesis (Brazert et al., 2020). And here we provide several lines of evidence demonstrating that ALA ameliorates the quality of oocytes induced by arsenite by suppressing oxidative stress-induced DNA damage and apoptosis, which would compromise the critical regulators involved in oocyte maturation and fertilization.

## Materials and methods

### Antibodies

Mouse monoclonal anti-γH_2_AX antibody were obtained from Abcam (Cambridge, MA, United States); Mouse monoclonal anti-α-tubulin FITC antibody, peanut agglutinin (PNA) -FITC, anti-actin antibody and anti-acetylation-α-tubulin (Lys-40) antibody were obtained from Sigma (St. Louis, MO, United States); Rabbit polyclonal anti-GAPDH antibody was obtained from Servicebio (Wuhan, China); Alexa Fluor 488-conjugated goat anti-mouse IgG (H + L) were obtained from Thermo Fisher Scientific (Waltham, MA, United States); HRP-labeled Goat Anti-Rabbit IgG (H + L) was obtained from Beyotime (Shanghai, China).

### Arsenite treatment and ALA supplementation

Arsenite and ALA were obtained from Sigma (St. Louis, MO, United States). The drugs were both dissolved and diluted in dimethyl sulfoxide (DMSO). Arsenite was further diluted into final working concentrations with *in vitro* medium (5 μM, 10 μM, 20 μM and 40 µM). ALA was further diluted into final working concentrations with *in vitro* medium (5 μM, 10 μM, 25 μM and 50 µM). The incubation medium should contain no more than 0.1% DMSO because of the cytotoxicity of DMSO itself. These doses were picked on the basis of data reported by previous examinations (Navarro et al., 2006; Makvandi et al., 2019).

### Experimental design

In this experiment, porcine oocytes were used as experimental model to explore the protective role of ALA against arsenite-induced meiosis defects since pigs share many physiological similarities with humans. The oocytes were randomly assigned to three groups as follows: 1) The control group; 2) The arsenite-treated group was supplemented with 40 µM arsenite; 3) The ALA-supplemented group was supplemented with 25 µM ALA. The concentration of arsenite (40 µM) and ALA (25 µM) was determined based on previous study and the effect of them on the first polar body extrusion. Each group contains at least 50 oocytes. The collected COCs were incubated in a preheated medium with arsenite and/or ALA and cultured in 38.5°C 5% CO_2_ incubator. After 30 h culture, oocytes developed to MI stage, and after 42 h of culture, oocytes developed to MII stage.

Experiment 1. The effects of ALA on the developmental competence and fertilization potential of arsenite-exposed porcine oocytes. The rates of the cumulus expansion of the COCs, the first polar body extrusion (PBE), fertilization and blastocyst were evaluated in control, arsenite-exposed and ALA-supplemented groups.

 Experiment 2. The effects of ALA on the nuclear maturation of arsenite-exposed oocytes. The spindle morphology, chromosomes alignment and microtubule stability were tested in control, arsenite-exposed and ALA-supplemented groups.

 Experiment 3. The effects of ALA on the cytoplasmic maturation of arsenite-exposed oocytes. The actin dynamics, localization of cortical granules and mitochondrial integrity were assessed in control, arsenite-exposed and ALA-supplemented groups. 

Experiment 4. The effects of ALA on the sperm binding ability of arsenite-exposed oocytes. The sperm binding to the zona pellucida were tested in control, arsenite-exposed and ALA-supplemented groups. 

Experiment 5. The molecular mechanism of ALA preventing the negative effect induced by arsenite. The level of ROS, DNA damage and apoptosis were determined in control, arsenite-exposed and ALA-supplemented groups.

### Porcine oocytes collection and *in vitro* maturation

To obtain high-quality oocytes, the ovaries were obtained and selected in slaughterhouses. The ovaries were conserved in 0.9% saline with penicillin (200,000 units) and streptomycin (250,000 units) at 38.5°C within 2 h. After washed for 3 times with fresh saline, the cumulus-oocyte complexes (COCs) were aspirated using a disposable syringe from the follicles on the outside layer of the ovaries. The COCs with a compact cumulus cells were selected and then moved into the maturation medium for the *in vitro* maturation. The maturation medium was made up on the basis of TCM-199 supplemented with 100 U/ml penicillin, 100 U/ml streptomycin, 0.5 μg/ml PG 600, 100 μl/ml of PFF, 50 μl/ml FBS, 10 ng/ml EGF and 0.1 mg/ml l-cysteine (Chen et al., 2021). COCs were moved into a drop of 50 µl maturation medium covered with mineral oil at 38.5°C and 5% CO_2_ for 28–30 h to metaphase I stage and for 42–44 h to metaphase II stage.

### Immunofluorescence staining and confocal microscopy

Denuded oocytes (DOs) were obtained by transferring COCs to 200 µl of 1 mg/ml hyaluronidase solution for 15 min to remove the granulosa cells. DOs were fixed in 4% paraformaldehyde (PFA) at room temperature for 20 min, then transferred into 0.5% Triton X-100 for 30 min for permeabilization. After blocking with blocking buffer (2% BSA-supplemented DPBS) for 1 h at room temperature, DOs were then incubated with primary antibody overnight at 4°C. Then the oocytes were incubated with secondary antibody for 1 h at room temperature after washed 3 times in DPBS. Then oocytes were counterstaining with propidium iodide (PI) or DAPI to stain nuclei for 10 min. Lastly, the samples were observed and imaged under a confocal microscope (Olympus, Japan).

### Western blot analysis

A total of 50 porcine oocytes for each group were collected and lysed in RIPA/6 x loading buffer (Beyotime Institute of Biotechnology, China) containing protease inhibitors, which heated at 95°C for 5 min. Proteins were separated on 6% future PAGE protein prep gel (Nanjing ACE Biotechnology Co., Ltd, China) and then transferred to polyvinylidene difluoride (PVDF) membranes. PVDF membranes containing the target protein were blocked in Tris-buffered saline Tween 20 (TBST) containing 5% skimmed milk powder for 3 h at room temperature to block up nonspecific sites. And then PVDF membranes were incubated with anti-acetylated α-tubulin antibody (1:8000) or anti-GAPDH antibody (1:4000) overnight at 4°C. After washed 3 times in TBST, the PVDF membranes were transferred into secondary antibodies and incubated for 2.5 h at room temperature. Chemiluminescence was performed with ECL Plus (New Cell & Molecular Biotech Co., Ltd, China) and signals were acquired by Chemiluminescence Imaging System 398 (Uvltec Ltd. Cambridge, United Kingdom).

### *In vitro* fertilization

Fresh semen from 20-month-old Landrace Pigs were diluted and resuspend in IVF medium to a final concentration of 0.25×10^6^ sperms/µl, followed by the oocytes were incubated at 38.5°C for 0.5 h. Then 15 MII oocytes and 50 µl sperms were incubated together in fertilization medium for a total of 5 h at 38.5°C, 5% CO_2_. After washed 3 times to remove the extra sperms, the oocytes were cultured in 4-well dishes with 400 µl of embryo medium at 38.5°C, 5% CO_2_. With the addition of sperm, the 2-cell embryo at 32 h was scored as successful fertilization. The blastocyst was observed and counted at 144 h to view the embryonic development.

### Sperm binding assay

Fresh semen were diluted and resuspend in IVF medium to a final concentration of 0.25×10^6^ sperms/µl, followed by the oocytes were incubated at 38.5°C for 0.5 h. Then 15 MII oocytes and 50 µl sperms were incubated together in fertilization medium for a total of 1 h at 38.5°C, 5% CO_2_. Sperm binding to oocytes were observed using capacitated sperm and two-cell embryos as a negative wash control. After the samples were fixed in 4% PFA for 20 min, sperm heads were stained with DAPI quantified the sperm binding capacity. The number of sperms bound to each oocyte was observed and counted by confocal microscopy. The results reflect the mean ± SEM from at least three independently obtained samples, each containing 10–15 porcine oocytes/embryos.

### Determination of mitochondrial distribution

Oocytes were incubated in the maturation medium containing 500 nM cell permeant MitoTracker Red CMXRos (Thermo Fisher, United States) to evaluate the distribution of active mitochondria. Oocytes should be incubated in 38.5°C 5% CO2 incubator for 30 min. Then the oocytes were washed four times with the maturation medium and observed under the confocal microscope (Olympus, Japan).

### Determination of ROS generation

To determine the levels of intracellular ROS production, oocytes were transferred into medium with the fluorescent probe [dichlorofluorescein (DCFH-DA)], according to the manufacturer’s instruction (Beyotime Institute of Biotechnology, China). The DCFH-DA working concentration were 10 μM and the incubation time were 30 min. Oocytes should be incubated in 38.5°C 5% CO2 incubator for 30 min. After washing three times in DPBS containing 0.3% PVP, the fluorescent intensity of the oocytes were observed under the confocal microscope (Olympus, Japan).

### Annexin-V staining

The oocytes apoptosis were detected with the Annexin-V-FITC apoptosis kit (Vazyme, China). Specifically, after washed three times in DPBS, the denuded oocytes were transferred into mixture containing Annexin-V-FITC and the binding buffer (1:9) for 30 min in the dark for the incubation. Then the oocytes were washed three times in DPBS and observed under the confocal microscope (Olympus, Japan).

### Statistical analysis

Data are representative of at least three independent repetitions, and data values are expressed as mean ± SEM. The number of oocytes observed is labeled in parentheses (n). The data were expressed as mean ± SEM and analyzed by one-way ANOVA, followed by LSD’s post hoc test, which was provided by SPSS16.0 statistical software. One-way ANOVA were respectively used for parametric and nonparametric variables. The level of significance was accepted as *p* < 0.05.

## Results

### ALA improves the developmental ability and fertilization capacity in arsenite-exposed oocytes

**FIGURE 1:**
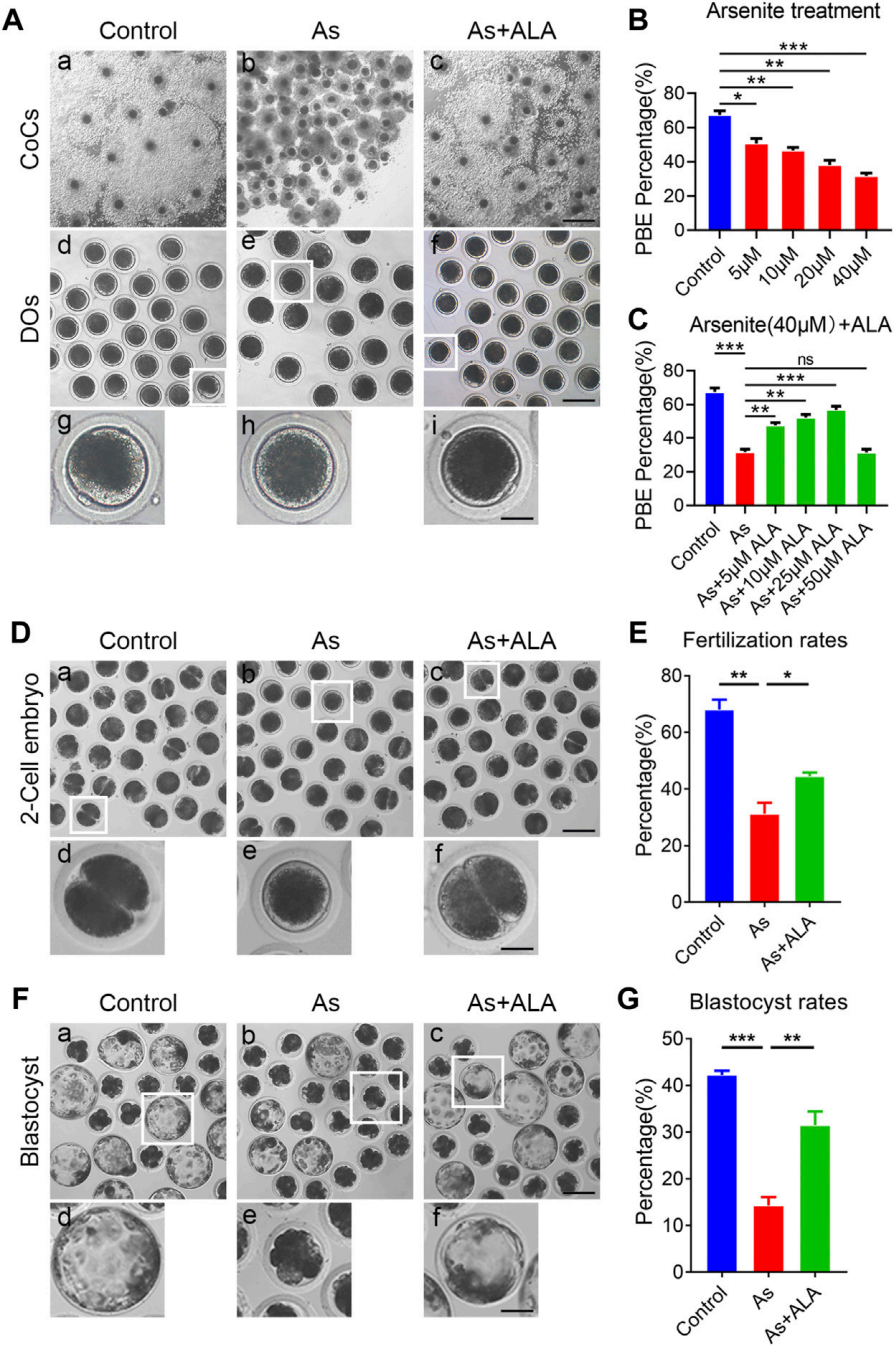
Effects of ALA supplementation on the porcine oocyte maturation and fertilization ability in arsenite-exposed oocytes. **(A)** Representative images of *in vitro* matured oocytes in control, arsenite-exposed and ALA-supplemented groups. Cumulus cell expansion of oocytes and PB1 extrusion were imaged by the inverted light microscope. Scale bar: 350 µm (a-c); 150 µm (d-f); 50 µm (g-i). **(B)** The rate of polar body extrusion were calculated in control and different concentrations of arsenite-exposed groups (5, 10, 20 and 40 µM) after culture for 44 h *in vitro*. **(C)** The rate of polar body extrusion were calculated in control, 40 µM arsenite-exposed and different concentrations of ALA-supplemented groups (5, 10, 25 and 50 µM) after *in vitro* maturation. **(D)** Representative images of 2-cell embryos in control, arsenite-exposed and ALA-supplemented groups. Scale bar: 120 µm (a-c); 40 µm (d-f). **(E)** The rate of *in vitro* fertilization were calculated in control, arsenite-exposed and ALA-supplemented groups. **(F)** Representative images of blastocysts developed from the fertilized control, arsenite-exposed and ALA-supplemented groups. Scale bar: 150 µm (a-c); 60 µm (d-f). **(G)** The rate of blastocysts were calculated in control, arsenite-exposed and ALA-supplemented groups. Data were presented as mean percentage (mean ± SEM) of three independent experiments. *p < 0.05, **p < 0.01, ***p < 0.001.

To investigate the potential protective role of ALA in the improvement of meiotic competence of porcine oocytes, we first examined the cumulus expansion of the COCs and the occurrence of first polar body extrusion (PBE) of oocytes, both are essential for meiotic maturation and developmental competence (Yin et al., 2019). For this purpose, different concentrations of arsenite (5, 10, 20 and 40 µM) and ALA (5, 10, 25 and 50 µM) were supplemented into the IVM medium, respectively. As shown in Figure 1A, most of the cumulus cells surrounding control oocytes were fully expanded, whereas those in the arsenite-exposed group were partially expanded or not expanded (Figure 1A). Adversely, supplementation of ALA is able to improve the cumulus expansion, especially the concentration of 25 μM, which is selected for the further studies. In addition, the majority of oocytes in the control group undergo normal PBE, but failed to do so following arsenite exposure (Figure 1A). Quantitative analysis showed a dose-dependent decrease in the proportion of PBE after arsenite treatment compared to the controls (control: 67.4 ± 2.4%, n = 137; 5 µM: 50.7 ± 2.9%, n = 120, *p* < 0.05; 10 µM: 46.6 ± 1.8%, n = 127, *p* < 0.01; 20 µM: 38.1 ± 2.7%, n = 123, *p* < 0.01; 40 µM: 31.7 ± 1.7%, n = 116, *p* < 0.001; Figure 1B), especially the concentration of 40 µM (67.4 ± 2.4%, n = 137 vs. 31.7 ± 1.7%, n = 116, *p* < 0.001; Figures 1A,C). Whereas, ALA increased the proportion of PBE of arsenite-exposed oocytes, especially the concentration of 25 µM (56.7 ± 2.2%, n = 201 vs. 31.7 ± 1.7%, n = 116, *p* < 0.001; Figures 1A,C). Therefore, we chose the concentration of 40 µM arsenite and 25 µM ALA for the follow-up studies because they affected the cumulus expansion of the COCs and the occurrence of PBE most effectively.

Fertilization capacity is also a key indicator reflecting oocytes quality, thus we further evaluated whether ALA could elevate the fertilization potential of oocytes exposed to arsenite (Gadella, 2012). The results showed that the majority of control oocytes were successfully fertilized and developed into two-cell embryos, whereas the fertilization rate of oocytes in the arsenite-exposed group remarkably decreased compared with the controls (31.2 ± 3.9%, n = 101 vs. 68.0 ± 3.5%, n = 159, *p* < 0.01; Figures 1D,E). As expected, ALA significantly improved fertilization rates of the oocyte exposed to arsenite (44.4 ± 1.4%, n = 108, *p* < 0.05; Figures 1D,E). We then further evaluated the subsequent early embryonic development of fertilized oocytes by examining the blastocyst rates. As shown in Figure 1F, the blastocyst rates of oocytes in arsenite-exposed group were significantly lower than that in the control group (14.3 ± 1.8%, n = 113 vs. 42.2 ± 0.9%, n = 125, *p* < 0.001; Figures 1F,G). As expected, ALA significantly increased the blastocyst rates of the arsenite-exposed oocytes (31.5 ± 2.9%, n = 142, *p* < 0.01; Figures 1F,G). The above results indicate that ALA had a beneficial effect on the fertilization ability of oocytes exposed to arsenite and promotes the subsequent embryonic developmental.

### ALA restores the spindle assembly, chromosome alignment and microtubule stability in arsenite-exposed oocytes

**FIGURE 2:**
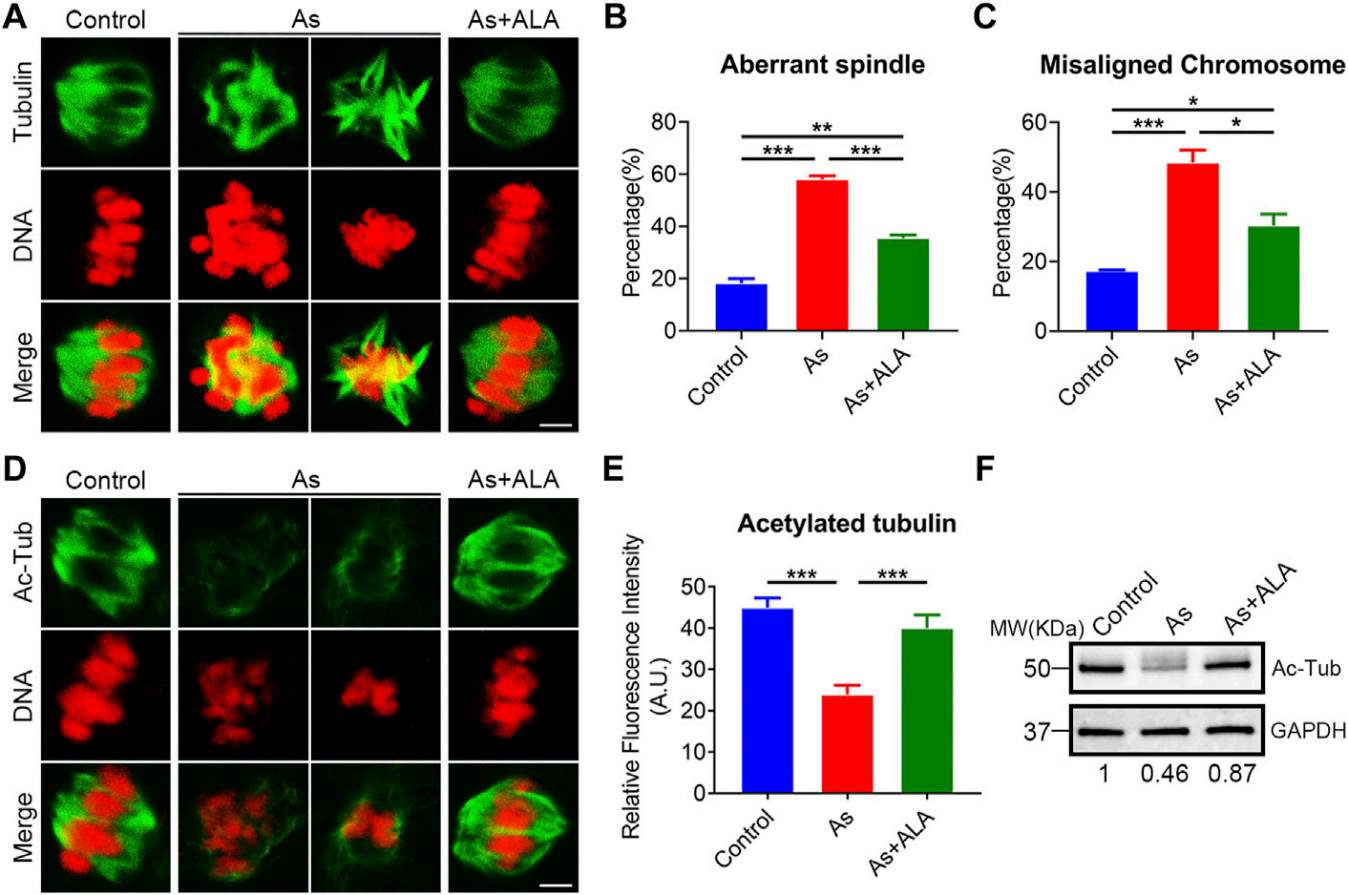
Effect of ALA supplementation on spindle assembly, chromosome alignment and microtubule stability in arsenite-exposed oocytes. **(A)** Representative images of spindle morphologies and chromosome alignment in control, arsenite-exposed and ALA-supplemented groups. MI oocytes were immunostained with anti-α-tubulin-FITC antibody and counterstained with propidium iodide (PI). Scale bar, 3 µm. **(B)** The rate of aberrant spindle were calculated in control, arsenite-exposed and ALA-supplemented groups. **(C)** The rate of misaligned chromosomes were calculated in control, arsenite-exposed and ALA-supplemented groups. **(D)** Representative images of acetylated α-tubulin in control, arsenite-exposed and ALA-supplemented groups. MI oocytes were immunostained with anti-acetyl-α-tubulin antibody and counterstained with propidiumiodide (PI). Scale bar, 3 µm. **(E)** The fluorescence intensity of acetylated α-tubulin was quantified in control, arsenite-exposed and ALA-supplemented groups. **(F)** The acetylation level of α-tubulin were measured by immunoblotting in control, arsenite-exposed and ALA-supplemented groups. The blots were probed with anti-acetyl-α-tubulin and anti-GAPDH antibodies, respectively. Data were presented as mean percentage (mean ± SEM) of three independent experiments. *p < 0.05, **p < 0.01, ***p < 0.001.

Studies reported that meiotic arrest is frequently accompanied with the impairment of cytoskeleton structures and functions (Zhou et al., 2019). We thus examined the spindle morphology and chromosomes alignment in oocytes following arsenite and ALA treatment. For this purpose, oocytes were immunostained with anti-α-tubulin-FITC antibodies to visualize the spindle morphology with PI to observe the chromosome alignment. The results showed that, oocytes displayed a typical barrel-like spindle apparatus with a well-aligned chromosome on the equatorial plate in the control group. In sharp contrast, various types of aberrant spindle morphologies with misaligned chromosomes were observed in arsenite-exposed oocytes (spindle: 18.2 ± 1.7%, n = 191 vs. 58.0 ± 1.4%, n = 143, *p* < 0.001; chromosome: 17.3 ± 0.3%, n = 229 vs. 48.5 ± 3.6%, n = 162, *p* < 0.001; Figures 2A–C). As expected, ALA significantly decreased the incidence of abnormal structure of spindle/chromosome (spindle: 35.6 ± 1.1%, n = 163, *p* < 0.001; chromosome: 30.3 ± 3.3%, n = 155, *p* < 0.05; Figures 2A–C).

Normally, structural abnormalities of spindle were frequently associated with the disruption of acetylation level of α-tubulin, an indicator of stable microtubules (Fernandez-Barrera and Alonso, 2018). We then test the acetylation level of α-tubulin porcine oocyte following arsenite and ALA treatment. To this end, oocytes were immunostained with anti-acetylated-tubulin-FITC antibody to observe the acetylation level of α-tubulin with PI to analyze the chromosome alignment. The results showed that the fluorescence intensity of acetylation level of α-tubulin was significantly reduced in the arsenite-exposed oocytes compared to the control group (23.9 ± 2.3, n = 23 vs. 45.0 ± 2.4, n = 23, *p* < 0.001; Figures 2D,E), but significantly raised following ALA supplementation by contrast (40.0 ± 3.2, n = 14 vs. 23.9 ± 2.3, n = 23, *p* < 0.001; Figures 2D,E). This observations were further confirmed with western blotting analysis (Figure 2F). Above all, these results indicate that ALA supplementation restore the spindle structure impaired by arsenite probably through the maintenance of acetylation level of α-tubulin.

### ALA restores the actin dynamics in arsenite-exposed oocytes

**FIGURE 3:**
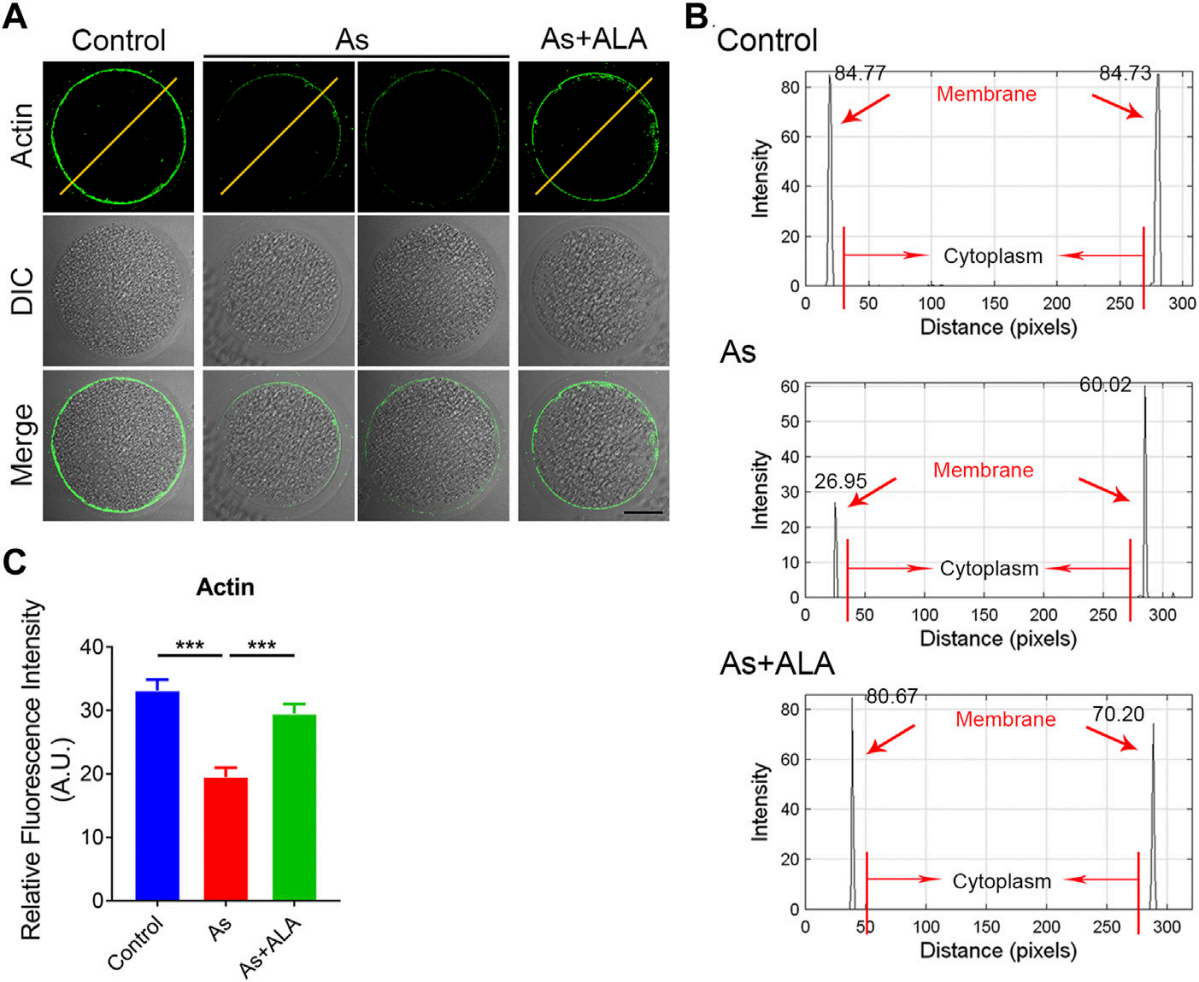
Effect of ALA supplementation on actin dynamics in arsenite-exposed oocytes. **(A)** Representative images of the localization of microfilaments in control, arsenite-exposed and ALA-supplemented groups. Scale bar, 30 µm. **(B)** The graphs showed the fluorescence intensity profiling of actin filaments control, arsenite-exposed and ALA-supplemented groups. Pixel intensities were measured along the lines which were drawn across the oocytes. **(C)** The fluorescence intensity of actin signals in control, arsenite-exposed and ALA-supplemented groups. Data were presented as mean percentage (mean ± SEM) of three independent experiments. ***p < 0.001.

Actin assembly is crucial for spindle assembly and cortical polarization during meiosis (Stricker et al., 2010). Thus, we next assessed the actin dynamics following arsenite and ALA treatment. To this end, anti-actin antibody was used to label the F-actin. As shown in Figure 3A, actin filaments of oocytes from the control group were concentrated uniformly on the plasma membrane with robust signals. Nevertheless, the accumulation of actin signals on the plasma membrane was diminished or completely lost in arsenite-exposed oocytes. Quantitative analysis showed that actin signals were significantly decreased in arsenate-exposed oocytes in comparison to the controls, but significantly increased following ALA supplementation within expectation (control: 33.1 ± 1.7, n = 20, *p* < 0.001; Arsenite: 19.5 ± 1.5, n = 21; ALA: 29.5 ± 1.5, n = 21, *p* < 0.001; Figures 3B,C), indicating the impaired actin dynamics induced by arsenite could be recovered by ALA.

### ALA recovers the localization of cortical granules and mitochondrial integrity in arsenite-exposed oocytes

**FIGURE 4:**
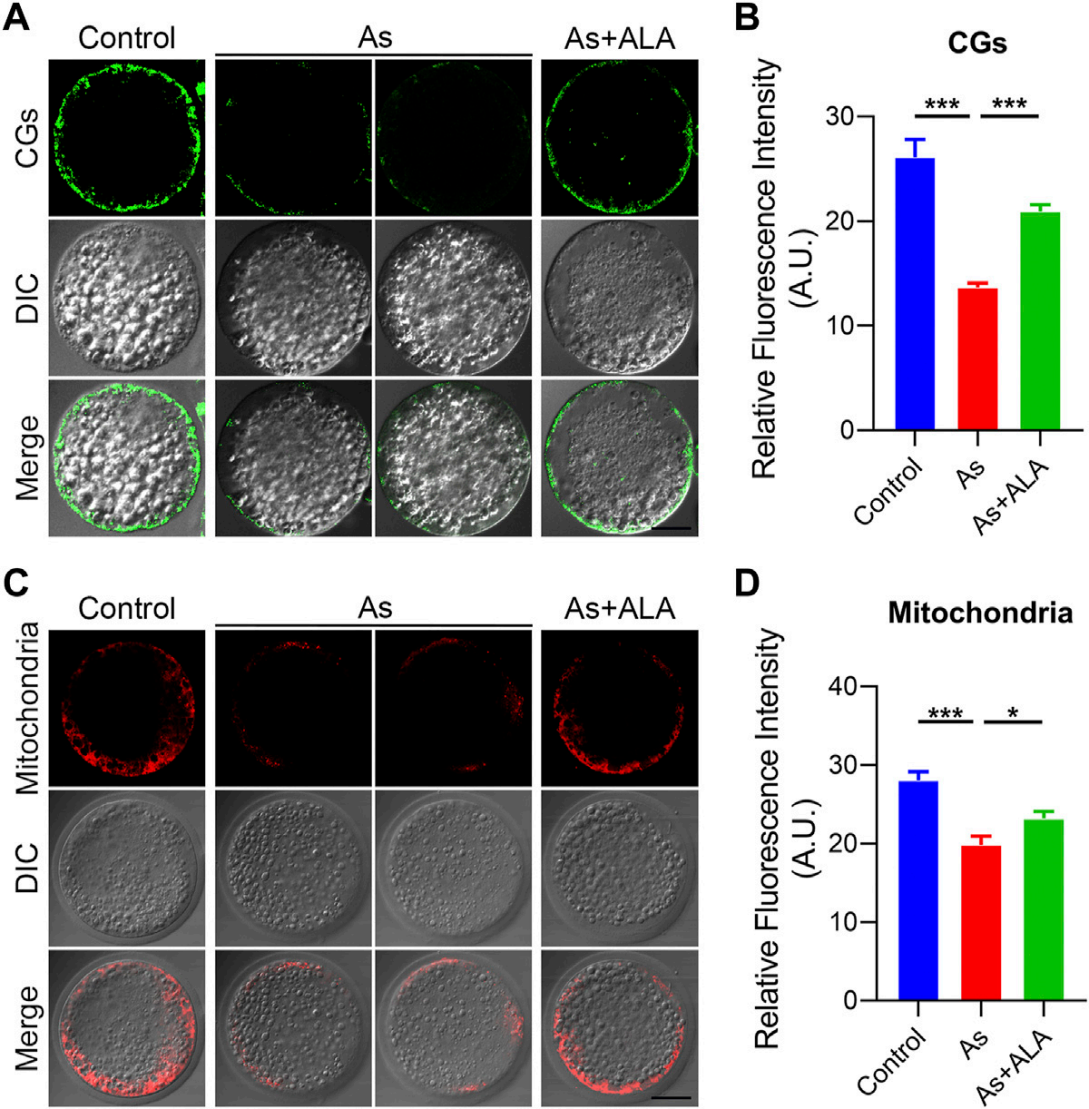
Effect of ALA supplementation on the localization of cortical granules and mitochondrial integrity in arsenite-exposed oocytes. **(A)** Representative images of the localization of cortical granules in control, arsenite-exposed and ALA-supplemented groups. LCA-FITC was used to show the CGs in the oocytes. Scale bar, 30 µm. **(B)** The fluorescence intensity of cortical granules in control, arsenite-exposed and ALA-supplemented groups. **(C)** Representative images of the mitochondrial integrity in control, arsenite-exposed and ALA-supplemented groups. Scale bar, 30 µm. **(D)** The fluorescence intensity of mitochondrial signals in control, arsenite-exposed and ALA-supplemented groups. Data were presented as mean percentage (mean ± SEM) of three independent experiments. *p < 0.05, ***p < 0.001.

Cortical granules (CGs) exocytosis and mitochondrial dynamics are considered to be two important indicators of oocyte cytoplasmic maturation (Wang et al., 2019). Following fertilization, the cortical granules releases the contents into the extracellular space by exocytosis to prevent multiple sperm from entering the oocytes (Santella et al., 2020; Rojas et al., 2021). Thus, we then examine whether ALA would rescue the dynamics of CGs by staining with its marker PNA-FITC. As shown in Figure 4A, CGs were localized in the subcortical region of the oocytes uniformly and continuously in the control group. In contrast, arsenite destroy the normal distribution pattern of CGs by showing the discontinued or completely disappeared signals. Quantitative analysis revealed that the fluorescence signal of CGs of the arsenite-exposed oocytes were significantly lower than that of the control oocytes, but was markedly increased following ALA supplementation (control: 26.1 ± 1.7, n = 28, *p* < 0.001; Arsenite: 13.6 ± 0.4, n = 35; ALA: 20.9 ± 0.6, n = 11, *p* < 0.001; Figure 4B).

It requires mitochondria to provide a large amount of ATP during oocyte maturation and fertilization processes (Burke, 2017). Studies report that the impaired oogenesis and embryogenesis are highly correlated with mitochondrial dysfunction (Babayev and Seli, 2015). Thus, we further examined the effect of ALA on the mitochondrial integrity of the arsenite-exposed oocytes by staining with MitoTracker. In the control group, mitochondria were accumulated around lipid droplets in the subcortical region of porcine oocytes (Figure 4C), but lost the specific localization in arsenite-exposed oocytes. Quantitative analysis revealed that the fluorescence intensity of the mitochondrial signal was significantly reduced in the arsenite-exposed group compared to the controls (19.8 ± 1.1, n = 40 vs. 28.0 ± 1.1, n = 26, *p* < 0.001; Figure 4D), and rescued by the ALA supplementation as expected (23.2 ± 0.9, n = 26, *p* < 0.05; Figure 4D). Taken together, the above results indicate ALA rescued the impairment of cytoplasmic maturation including the cortical granules and mitochondria of oocytes exposed to arsenite.

### ALA rescues the sperm binding to the zona pellucida in arsenite-exposed oocytes

**FIGURE 5:**
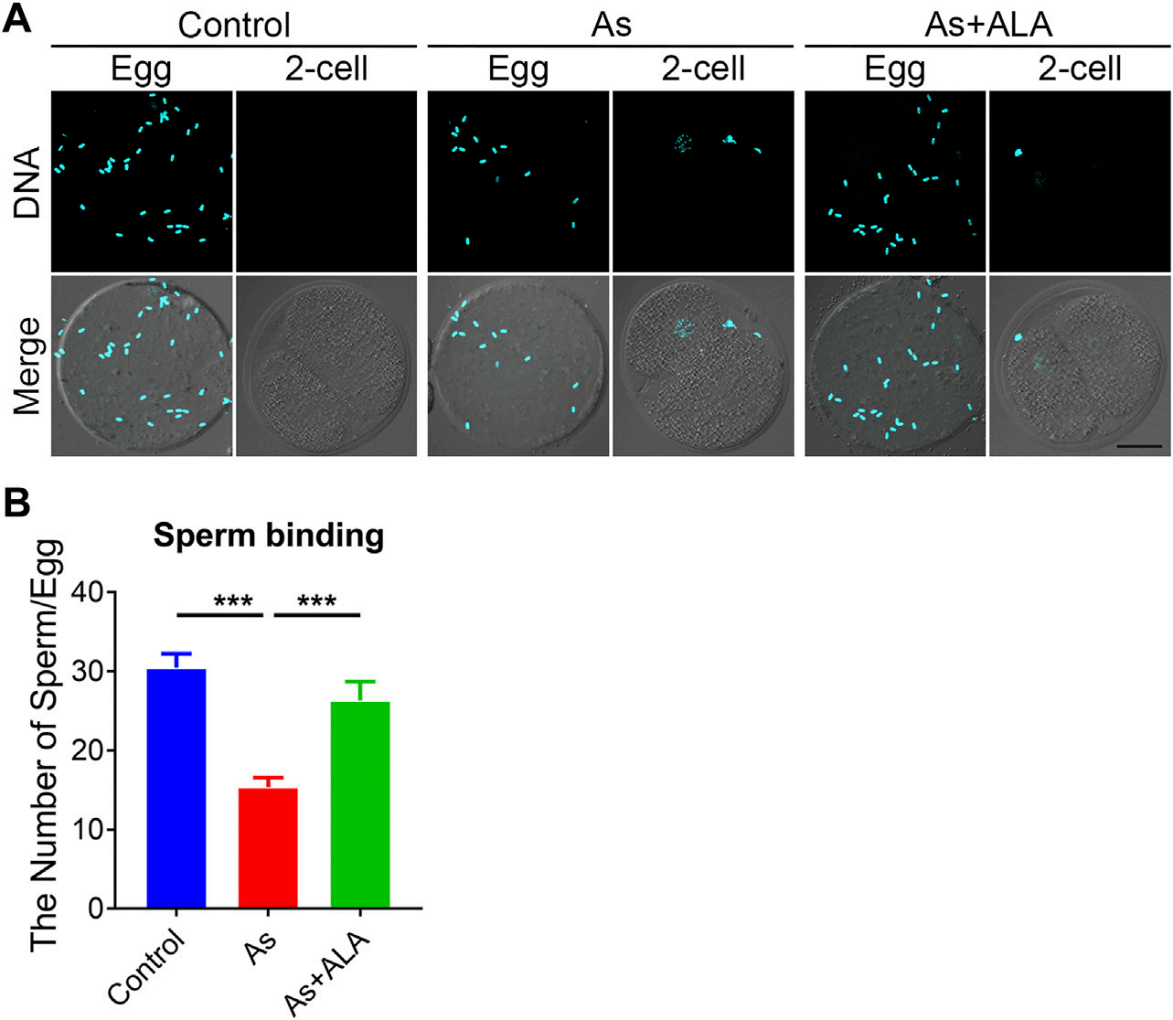
Effect of ALA supplementation sperm binding to the zona pellucida in arsenite-exposed oocytes. **(A)** Eggs and 2-cell embryos in control, arsenite-exposed and ALA-supplemented groups were incubated (1 h) with capacitated sperm. After washing with a wide-bore pipette to remove all but two to six sperm on normal two-cell embryos (negative control), eggs and embryos were stained with DAPI. Scale bar, 30 µm. **(B)** The number of sperms binding to the zona pellucida were calculated in control, arsenite-exposed and ALA-supplemented groups. Data were presented as mean percentage (mean ± SEM) of three independent experiments. ***p < 0.001.

In mammals, successful fertilization depends on series of sperm-egg interaction events (Dai et al., 2017). From the begining, the sperm binds to the extracellular matrix around the zona pellucida and only by successfully crossing the zona pellucida can it successfully fuse with the oocyte membrane to form a fertilized egg (Oliveira-Araujo et al., 2020). To evaluated whether ALA could rescue the sperm binding ability and elevate the fertilization potential, a sperm-oocyte binding assay was performed. The sperm heads were stained with Hoechst to count the number of sperm bound to the zona pellucida. The unfertilized eggs and two-cell embryos were used as a negative control. As shown in Figure 5A, in the two-cell embryos, the zona pellucida no longer supported additional sperm binding following fertilization, because of the loss of the sperm binding site. In the arsenite-exposed oocytes, the number of sperm binding to the zona pellucida was remarkably reduced compared with the controls (15.3 ± 1.2, n = 29 vs. 30.4 ± 1.8, n = 39, *p* < 0.001; Figures 5A,B), but this reduction was partially restored by ALA supplementation (26.3 ± 2.4, n = 33, *p* < 0.001; Figures 5A,B).

### ALA reduces ROS level and DNA damage to suppress apoptosis in arsenite-exposed oocytes

**FIGURE 6:**
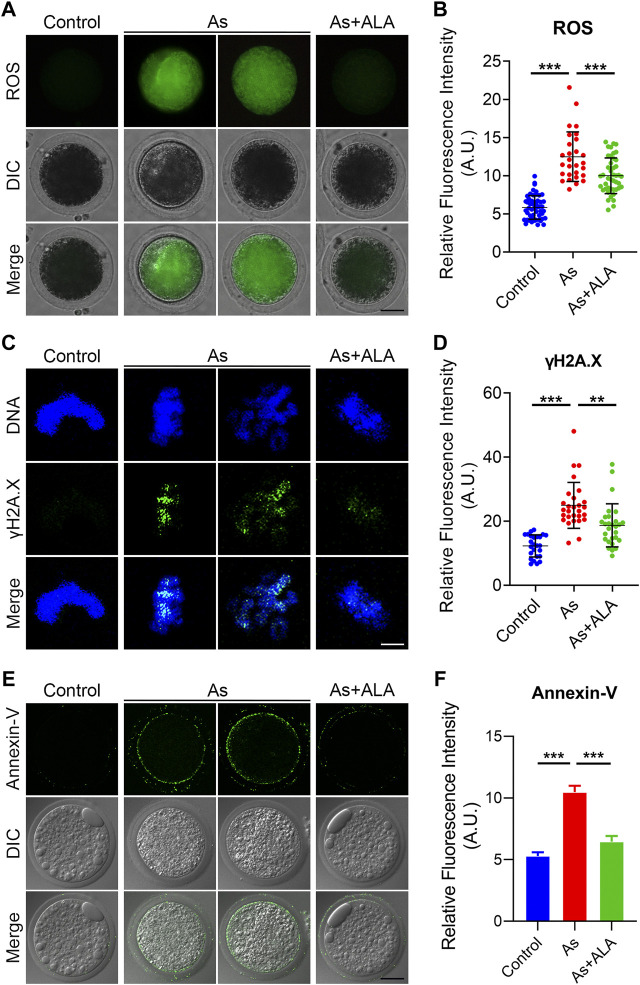
Effect of ALA supplementation on ROS levels, DNA damage and apoptosis in arsenite-exposed oocytes. **(A)** Representative images of ROS levels in control, arsenite-exposed and ALA-supplemented groups. Scale bar, 30 µm **(B)** The fluorescence intensity of ROS were measured in control, arsenite-exposed and ALA-supplemented groups. **(C)** Representative images of DNA damage in control, arsenite-exposed and ALA-supplemented groups. Oocytes were immunostained with annexin-V-FITC. Scale bar, 2.5 µm. **(D)** The fluorescence intensity of γH2AX signals were measured in control, arsenite-exposed and ALA-supplemented groups. **(E)** Representative images of apoptotic oocytes in control, arsenite-exposed and ALA-supplemented groups. Oocytes showing signals of fluorescence (green) in their membrane are regarded as early-stage apoptosis. Scale bar, 30 µm. **(F)** The fluorescence intensity of Annexin-V signals were measured in control, arsenite-exposed and ALA-supplemented groups. Data were presented as mean percentage (mean ± SEM) of three independent experiments. *p < 0.05, ***p < 0.001.

Previous studies showed that arsenite has a certain oxidizing effect, leading to the increased level of ROS in cells of plants and animals (Srinivas et al., 2019). The accumulation of ROS further damage the DNA structures and leads to premature apoptosis and thus disrupts the normal functional development of oocytes (Zhang et al., 2020). More importantly, ALA has been reported to be able to reduce oxidative stress to some extent in experiments on mice and human somatic cells (Hiller et al., 2016). Thus, we hypothesize that arsenite leads to the deterioration and degradation of oocytes quality during maturation and fertilization process, and ALA could prevent the negative effect induced by arsenite in oocytes by inhibiting the ROS level, DNA damage and apoptosis. To verify this hypotheses, we measured and compared the ROS level of oocytes between the arsenite-exposed group and ALA-supplemented group with DCFH-DA Staining. The results showed that the level of ROS in arsenite-exposed oocytes was much higher than controls (12.5 ± 0.6, n = 28 vs. 5.8 ± 0.2, n = 52, *p* < 0.001; Figures 6A,B), and effectively decreased following ALA supplementation (10.0 ± 0.4, n = 44, *p* < 0.001; Figures 6A,B).

Since the excessive oxidative stress tend to inhibit the self-repair of double-stranded DNA, leading to the massive DNA damage and consequent apoptosis (Pisoschi and Pop, 2015; Senoner and Dichtl, 2019). Thus, we assessed the DNA damage by determining the fluorescence intensity by immunofluorescence staining using the anti-γH2AX antibody. As shown in Figure 6C, the fluorescence intensity of γH2AX signals was significantly increased compared with the controls (24.9 ± 1.4, n = 28 vs. 12.3 ± 0.7, n = 25, *p* < 0.001; Figures 6C,D). As expected, the supplementation of ALA decreased the occurrence of DNA damage (18.7 ± 1.3, n = 28, *p* < 0.01; Figures 6C,D). Meanwhile, we observed a strong fluorescent signal of Annexin-V on the plasma membrane of the arsenite-exposed oocytes, while the fluorescent signal were almost undetectable or very weak in the controls (Figure 6E). The quantitative results indicated that the fluorescence intensity of Annexin-V was significantly increased in comparison to the controls (10.5 ± 0.5, n = 25 vs. 5.3 ± 0.3, n = 25, *p* < 0.001; Figures 6E,F), but decreased following ALA supplementation (6.4 ± 0.5, n = 26, *p* < 0.001; Figures 6E,F). Taken together, the above observations indicate that the apoptotic oocytes caused by high level of ROS and DNA damage in arsenite-exposed oocytes could be rescued following ALA supplementation.

## Discussion

Female fertility is regulated by both the female reproductive system and the endocrine system (Nicolopoulou-Stamati and Pitsos, 2001; Duursen et al., 2020). Arsenite, a most well-known endocrine disrupting chemicals, was reported to cause the accumulation of ROS and reactive nitrogen species (RNS) (Davey et al., 2008). Excessive ROS and RNS attack important macromolecules and organelles leading to cell apoptosis (Kaminskyy and Zhivotovsky, 2014; Tapeinos et al., 2018). As a strong antioxidant naturally present in the body, we hypothesize that ALA could restores the meiotic competency and fertilization capacity of porcine oocytes induced by arsenite. To verify this hypothesis and investigate the potential mechanisms, we use porcine oocytes as an experimental model. Here we aim to provide a new perspective for the application of ALA in preventing the declined oocyte quality induced by environmental EDCs.

The cumulus expansion of the COCs and the proportion of PBE are two key indicators of the porcine oocytes developmental competence (Lan et al., 2020; Du et al., 2021). Our results revealed that oocytes exhibited a lower proportion of PBE with impaired expansion of cumulus cells following arsenite treatment, indicating that arsenite impairs the oocyte meiotic maturation and developmental competence. As expected, the supplementation of ALA declined the PBE rates, suggesting that ALA indeed has the potential to ameliorate the oocytes developmental competence. To further reveal weather ALA could restore the arsenite-induced decline of oocyte fertilization capacity, we then calculated the fertilization rates and blastocyst development rates of oocytes. As expected, the supplementation of ALA improved the fertilization rates and blastocyst development rates. This result suggested that ALA indeed has the potential to ameliorate the oocyte fertilization capacity of arsenite-exposed oocytes.

To further reveal the potential mechanisms of ALA to ameliorate the declined oocytes developmental competence induced by arsenite, we next examined the critical biological events occurred during the nuclear and cytoplasmic maturation processes of oocytes. The spindle assembly and function are intimately linked to the intrinsic dynamics of microtubules, which is responsible for the accurate chromosome segregation during meiosis (Brieno-Enriquez and Cohen, 2015). Also, Spindle assembly is a crucial event for euploidy during oocyte maturation. And our current study illustrated ALA supplementation restores the defected meiotic spindle morphology and chromosome misalignment caused by arsenite. Moreover, the disruption and loss of meiotic spindle assembly is always associated with the microtubule dynamics and stability (Cleary and Hancock, 2021). Tubulin acetylation that occurs on Lys-40 of the α-tubulin subunit is found both in somatic cells and oocytes as an indicator of stabilized microtubules. Our findings further confirm these phenomena and revealed that ALA could maintain the microtubule stability and thus to ameliorate the defected spindle/chromosome structures in the Arsnite-exposed oocytes.

Actin filaments (F-actin) occur as microfilaments, which is essential component of the cytoskeleton (Duan et al., 2020). Actin cytoskeleton dynamics are critical for cellular processes during oocyte meiotic maturation, including nuclear positioning, GVBD, spindle migration, spindle rotation, chromosome segregation, and PBE (Duan and Sun, 2019). Our findings confirmed that the notably distroyed actin cytoskeleton dynamics caused by arsenite were prevented by the supplementation of ALA. The distribution and polymerization of F-actin could be another dominant mechanism by which ALA could restore the arsenite-induced oocyte meiotic failure.

Cortical granules are membrane-bound organelles located in the cortex of unfertilized oocytes, specifically exist in oocytes (Liu, 2011). Once fertilized, the cortical granules release its contents to cleave the sperm binding site ZP2, preventing polyspermy (Gahlay et al., 2010). Thus, proper dynamics of Cortical granules are often considered to be the key indicator of oocyte cytoplasmic maturation. Our datas indicated that ALA supplementation recovered the abnormal distribution of CGs induced by arsenite.

In addition, mitochondrial dynamics is another important sign of oocyte cytoplasmic maturation. Mitochondria provide the ATP for cellular activities during oocytes maturation and fertilization processes (Udagawa et al., 2014). And chromosomal segregation disorders, fertilization failures and early apoptosis are always paired with mitochondrial dysfunction (Seli et al., 2019). Our data revealed that ALA could protect the mitochondrial integrity destroyed by arsenite. These above observations confirmed that ALA could prevent the defective oocytes cytoplasmic maturation and to ameliorate the oocytes developmental competence destroyed by arsenite.

During fertilization, sperms bind to the zona pellucida and then undergo the acrosome reaction, after sperms penetrate the zona pellucida and finally fuse with the oocyte plasma membrane (Tokuhiro and Dean, 2018). So, if sperm do not bind, they cannot penetrate the zona matrix, and they cannot fuse with the plasma membrane. In addition, after fertilization, ZP2 is cleaved by ovastacin to ensure monospermic fertilization because polyspermic aneuploidy is also a significant threat to embryonic survival, and mice have developed strategies (Xiong et al., 2017). Prevention of sperm binding to the zona pellucida provides the ultimate postfertilization block to polyspermy (Vogt et al., 2019). Based on these understandings, our data found that arsenite impaired the ability of oocytes to bind sperms, and ALA supplementation is able to restore the declined numbers of sperm binding to the zona pellucida. This observation implied that ALA could restored sperm binding abilities and thus improve the fertilization potential impaired by arsenite.

Accumulating studies have indicated that oxidative stress induced by environmental endocrine disruptors might be a major cause for the deterioration of oocyte quality (Ding et al., 2020; Lombo and Herraez, 2021). Thus, we hypothesize that as a strong antioxidant, ALA supplementation could eliminate the excessive ROS, which ameliorate oocyte developmental competence and fertilization potential. Our results illustrated ALA supplementation attenuated ROS levels and thus inhibit DNA damage and apoptosis induced by arsenite.

**FIGURE 7:**
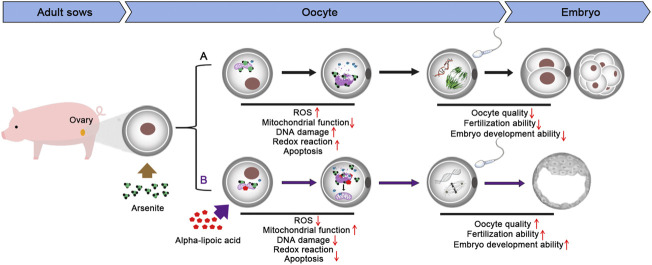
Diagram of the effects of ALA supplementation on arsenite-exposed oocytes.

Taken together, we provide a body of evidence that the supplementation of ALA restores, at least partially, all of the meiotic abnormalities and impaired fertilization ability induced by arsenite, through suppressing the increase of ROS level and the occurrence of DNA damage along with apoptosis caused by arsenite. Meanwhile, we demonstrate that ALA supplementation is an effective and feasible strategy to prevent the potential negative effect induced by environmental endocrine disruptors and ameliorate the oocyte quality (Figure 7).

## Data Availability

The raw data supporting the conclusions of this article will be made available by the authors, without undue reservation.

## References

[B1] Babayev E. Seli E. (2015). Oocyte mitochondrial function and reproduction. Curr. Opin. Obstet. Gynecol. 27 (3), 175–181. 10.1097/GCO.0000000000000164 25719756 PMC4590773

[B2] Barati E. Nikzad H. Karimian M. (2020). Oxidative stress and male infertility: Current knowledge of pathophysiology and role of antioxidant therapy in disease management. Cell. Mol. Life Sci. 77 (1), 93–113. 10.1007/s00018-019-03253-8 31377843 PMC11105059

[B3] Bourguignon N. S. Bonaventura M. M. Rodriguez D. Bizzozzero M. Ventura C. Nunez M. (2017). Evaluation of sodium arsenite exposure on reproductive competence in pregnant and postlactational dams and their offspring. Reprod. Toxicol. 69, 1–12. 10.1016/j.reprotox.2017.01.002 28077272

[B4] Brazert M. Kranc W. Nawrocki M. J. Sujka-Kordowska P. Konwerska A. Jankowski M. (2020). New markers for regulation of transcription and macromolecule metabolic process in porcine oocytes during *in vitro* maturation. Mol. Med. Rep. 21 (3), 1537–1551. 10.3892/mmr.2020.10963 32016446 PMC7002967

[B5] Brieno-Enriquez M. A. Cohen P. E. (2015). Double trouble in human aneuploidy. Nat. Genet. 47 (7), 696–698. 10.1038/ng.3344 26111508

[B6] Burke P. J. (2017). Mitochondria, bioenergetics and apoptosis in cancer. Trends Cancer 3 (12), 857–870. 10.1016/j.trecan.2017.10.006 29198441 PMC5957506

[B7] Chen J. Miao Y. Gao Q. Cui Z. Xiong B. (2021). Exposure to perfluorooctane sulfonate *in vitro* perturbs the quality of porcine oocytes via induction of apoptosis. Environ. Pollut. 284, 117508. 10.1016/j.envpol.2021.117508 34261219

[B8] Cleary J. M. Hancock W. O. (2021). Molecular mechanisms underlying microtubule growth dynamics. Curr. Biol. 31 (10), R560–R573. 10.1016/j.cub.2021.02.035 34033790 PMC8575376

[B9] Dai X. Lu Y. Zhang M. Miao Y. Zhou C. Cui Z. (2017). Melatonin improves the fertilization ability of post-ovulatory aged mouse oocytes by stabilizing ovastacin and juno to promote sperm binding and fusion. Hum. Reprod. 32 (3), 598–606. 10.1093/humrep/dew362 28137755

[B10] Davey J. C. Nomikos A. P. Wungjiranirun M. Sherman J. R. Ingram L. Batki C. (2008). Arsenic as an endocrine disruptor: Arsenic disrupts retinoic acid receptor-and thyroid hormone receptor-mediated gene regulation and thyroid hormone-mediated Amphibian tail metamorphosis. Environ. Health Perspect. 116 (2), 165–172. 10.1289/ehp.10131 18288313 PMC2235215

[B11] Di Nicuolo F. Castellani R. Ticconi C. Scambia G. Pontecorvi A. Di Simone N. (2021). α-Lipoic acid and its role on female reproduction. Curr. Protein Pept. Sci. 22 (11), 767–774. 10.2174/1389203722666211029102417 34719371

[B12] Ding N. Harlow S. D. Randolph J. F. Jr. Loch-Caruso R. Park S. K. (2020). Perfluoroalkyl and polyfluoroalkyl substances (pfas) and their effects on the ovary. Hum. Reprod. Update 26 (5), 724–752. 10.1093/humupd/dmaa018 32476019 PMC7456353

[B13] Du C. Davis J. S. Chen C. Li Z. Cao Y. Sun H. (2021). Fgf2/Fgfr signaling promotes cumulus-oocyte complex maturation *in vitro* . Reproduction 161 (2), 205–214. 10.1530/REP-20-0264 33434172

[B14] Duan X. Sun S. C. (2019). Actin cytoskeleton dynamics in mammalian oocyte meiosis. Biol. Reprod. 100 (1), 15–24. 10.1093/biolre/ioy163 30010726

[B15] Duan X. Li Y. Yi K. Guo F. Wang H. Wu P. H. (2020). Dynamic organelle distribution initiates actin-based spindle migration in mouse oocytes. Nat. Commun. 11 (1), 277. 10.1038/s41467-019-14068-3 31937754 PMC6959240

[B16] Duursen M. Boberg J. Christiansen S. Connolly L. Damdimopoulou P. Filis P. (2020). Safeguarding female reproductive health against endocrine disrupting chemicals-the freia Project. Int. J. Mol. Sci. 21 (9), E3215. 10.3390/ijms21093215 32370092 PMC7246859

[B17] Erickson N. Zafron M. Harding S. V. Marinangeli C. P. F. Rideout T. C. (2020). Evaluating the lipid-lowering effects of alpha-lipoic acid supplementation: A systematic review. J. Diet. Suppl. 17 (6), 753–767. 10.1080/19390211.2019.1651436 31416362

[B18] Fernandez-Barrera J. Alonso M. A. (2018). Coordination of microtubule acetylation and the actin cytoskeleton by formins. Cell. Mol. Life Sci. 75 (17), 3181–3191. 10.1007/s00018-018-2855-3 29947928 PMC11105221

[B19] Firrincieli A. Presentato A. Favoino G. Marabottini R. Allevato E. Stazi S. R. (2019). Identification of resistance genes and response to arsenic in rhodococcus aetherivorans Bcp1. Front. Microbiol. 10, 888. 10.3389/fmicb.2019.00888 31133997 PMC6514093

[B20] Gadella B. M. (2012). Dynamic regulation of sperm interactions with the zona pellucida prior to and after fertilisation. Reprod. Fertil. Dev. 25 (1), 26–37. 10.1071/RD12277 23244826

[B21] Gahlay G. Gauthier L. Baibakov B. Epifano O. Dean J. (2010). Gamete recognition in mice depends on the cleavage status of an egg's zona pellucida protein. Science 329 (5988), 216–219. 10.1126/science.1188178 20616279 PMC3272265

[B22] Gao Z. Tang X. Ye M. Gul I. Chen H. Yan G. (2021). Effects of silicon on the uptake and accumulation of arsenite and dimethylarsinic acid in rice (oryza sativa L.). J. Hazard. Mater. 409, 124442. 10.1016/j.jhazmat.2020.124442 33168309

[B23] Green M. P. Harvey A. J. Finger B. J. Tarulli G. A. (2021). Endocrine disrupting chemicals: Impacts on human fertility and fecundity during the peri-conception period. Environ. Res. 194, 110694. 10.1016/j.envres.2020.110694 33385395

[B24] Guidarelli A. Fiorani M. Cerioni L. Scotti M. Cantoni O. (2017). Arsenite induces DNA damage via mitochondrial ros and induction of mitochondrial permeability transition. Biofactors 43 (5), 673–684. 10.1002/biof.1375 28703385

[B25] Hiller S. DeKroon R. Hamlett E. D. Xu L. Osorio C. Robinette J. (2016). Alpha-lipoic acid supplementation protects enzymes from damage by nitrosative and oxidative stress. Biochim. Biophys. Acta 1860, 36–45. 10.1016/j.bbagen.2015.09.001 26344063 PMC5293714

[B26] Kaminskyy V. O. Zhivotovsky B. (2014). Free radicals in cross talk between autophagy and apoptosis. Antioxid. Redox Signal. 21 (1), 86–102. 10.1089/ars.2013.5746 24359220

[B27] Lan M. Zhang Y. Wan X. Pan M. H. Xu Y. Sun S. C. (2020). Melatonin ameliorates ochratoxin a-induced oxidative stress and apoptosis in porcine oocytes. Environ. Pollut. 256, 113374. 10.1016/j.envpol.2019.113374 31672358

[B28] Liu M. (2011). The Biology and dynamics of mammalian cortical granules. Reprod. Biol. Endocrinol. 9, 149. 10.1186/1477-7827-9-149 22088197 PMC3228701

[B29] Lombo M. Herraez P. (2021). The effects of endocrine disruptors on the male germline: An intergenerational health risk. Biol. Rev. Camb. Philos. Soc., 96 (4), 1243–1262. 10.1111/brv.12701 33660399

[B30] Makvandi A. Kowsar R. Hajian M. Mahdavi A. H. Tanhaei Vash N. Nasr-Esfahani M. H. (2019). Alpha lipoic acid reverses the negative effect of lps on mouse spermatozoa and developmental competence of resultant embryos *in vitro* . Andrology 7 (3), 350–356. 10.1111/andr.12596 30786163

[B31] Navarro P. A. Liu L. Ferriani R. A. Keefe D. L. (2006). Arsenite induces aberrations in meiosis that can Be prevented by coadministration of N-acetylcysteine in mice. Fertil. Steril. 85, 1187–1194. 10.1016/j.fertnstert.2005.08.060 16616091

[B32] Nicolopoulou-Stamati P. Pitsos M. A. (2001). The impact of endocrine disrupters on the female reproductive system. Hum. Reprod. Update 7 (3), 323–330. 10.1093/humupd/7.3.323 11392379

[B33] Oliveira-Araujo M. S. Lopes J. T. Nunes L. T. Almeida-Monteiro P. S. Nascimento R. V. D. Pereira V. A. (2020). Determination of the ideal volume of activating solution and the optimal ratio of spermatozoa per oocyte for Prochilodus brevis fertilization. Zygote 28 (2), 103–108. 10.1017/S0967199419000728 31735199

[B34] Palma-Lara I. Martinez-Castillo M. Quintana-Perez J. C. Arellano-Mendoza M. G. Tamay-Cach F. Valenzuela-Limon O. L. (2020). Arsenic exposure: A public health problem leading to several cancers. Regul. Toxicol. Pharmacol. 110, 104539. 10.1016/j.yrtph.2019.104539 31765675

[B35] Pisoschi A. M. Pop A. (2015). The role of antioxidants in the chemistry of oxidative stress: A review. Eur. J. Med. Chem. 97, 55–74. 10.1016/j.ejmech.2015.04.040 25942353

[B36] Prathima P. Pavani R. Sukeerthi S. Sainath S. B. (2018). α-Lipoic acid inhibits testicular and epididymal oxidative damage and improves fertility efficacy in arsenic-intoxicated rats. J. Biochem. Mol. Toxicol. 32 (2), e22016. 10.1002/jbt.22016 29214690

[B37] Quansah R. Armah F. A. Essumang D. K. Luginaah I. Clarke E. Marfoh K. (2015). Association of arsenic with adverse pregnancy outcomes/infant mortality: A systematic review and meta-analysis. Environ. Health Perspect. 123 (5), 412–421. 10.1289/ehp.1307894 25626053 PMC4421764

[B38] Rojas J. Hinostroza F. Vergara S. Pinto-Borguero I. Aguilera F. Fuentes R. (2021). Knockin' on egg's door: Maternal control of egg activation that influences cortical granule exocytosis in animal species. Front. Cell Dev. Biol. 9, 704867. 10.3389/fcell.2021.704867 34540828 PMC8446563

[B39] Salehi B. Berkay Yilmaz Y. Antika G. Boyunegmez Tumer T. Fawzi Mahomoodally M. Lobine D. (2019). Insights on the use of alpha-lipoic acid for therapeutic purposes. Biomolecules 9 (8), E356. 10.3390/biom9080356 31405030 PMC6723188

[B40] Santella L. Limatola N. Chun J. T. (2020). Cellular and molecular aspects of oocyte maturation and fertilization: A perspective from the actin cytoskeleton. Zool. Lett. 6, 5. 10.1186/s40851-020-00157-5 PMC7158055 32313685

[B41] Seli E. Wang T. Horvath T. L. (2019). Mitochondrial unfolded protein response: A stress response with implications for fertility and reproductive aging. Fertil. Steril., 111 (2), 197–204. 10.1016/j.fertnstert.2018.11.048 30691623

[B42] Senoner T. Dichtl W. (2019). Oxidative stress in cardiovascular diseases: Still a therapeutic target? Nutrients, 11 (9), E2090. 10.3390/nu11092090 31487802 PMC6769522

[B43] Solmonson A. DeBerardinis R. J. (2018). Lipoic acid metabolism and mitochondrial redox regulation. J. Biol. Chem. 293 (20), 7522–7530. 10.1074/jbc.TM117.000259 29191830 PMC5961061

[B44] Souza A. C. F. Ervilha L. O. G. Coimbra J. L. P. Bastos D. S. S. Guimaraes S. E. F. Machado-Neves M. (2020). Reproductive disorders in female rats after prenatal exposure to sodium arsenite. J. Appl. Toxicol. 40 (2), 214–223. 10.1002/jat.3897 31429093

[B45] Srinivas U. S. Tan B. W. Q. Vellayappan B. A. Jeyasekharan A. D. (2019). Ros and the DNA damage response in cancer. Redox Biol. 25, 101084. 10.1016/j.redox.2018.101084 30612957 PMC6859528

[B46] Stricker J. Falzone T. Gardel M. L. (2010). Mechanics of the F-actin cytoskeleton. J. Biomech. 43 (1), 9–14. 10.1016/j.jbiomech.2009.09.003 19913792 PMC2813332

[B47] Tapeinos C. Larranaga A. Sarasua J. R. Pandit A. (2018). Functionalised collagen spheres reduce H2o2 mediated apoptosis by scavenging overexpressed ros. Nanomedicine 14 (7), 2397–2405. 10.1016/j.nano.2017.03.022 28552642

[B48] Tokuhiro K. Dean J. (2018). Glycan-independent gamete recognition triggers egg zinc sparks and Zp2 cleavage to prevent polyspermy. Dev. Cell 46 (5), 627–640 e5. 10.1016/j.devcel.2018.07.020 30122633 PMC6549238

[B49] Tryndyak V. P. Borowa-Mazgaj B. Steward C. R. Beland F. A. Pogribny I. P. (2020). Epigenetic effects of low-level sodium arsenite exposure on human liver heparg cells. Arch. Toxicol. 94 (12), 3993–4005. 10.1007/s00204-020-02872-6 32844245

[B50] Udagawa O. Ishihara T. Maeda M. Matsunaga Y. Tsukamoto S. Kawano N. (2014). Mitochondrial fission factor Drp1 maintains oocyte quality via dynamic rearrangement of multiple organelles. Curr. Biol. 24 (20), 2451–2458. 10.1016/j.cub.2014.08.060 25264261

[B51] Vogt E. J. Tokuhiro K. Guo M. Dale R. Yang G. Shin S. W. (2019). Anchoring cortical granules in the cortex ensures trafficking to the plasma membrane for post-fertilization exocytosis. Nat. Commun. 10 (1), 2271. 10.1038/s41467-019-10171-7 31118423 PMC6531442

[B52] Wang F. Zhou X. Liu W. Sun X. Chen C. Hudson L. G. (2013). Arsenite-induced ros/rns generation causes zinc loss and inhibits the activity of poly(adp-ribose) polymerase-1. Free Radic. Biol. Med. 61, 249–256. 10.1016/j.freeradbiomed.2013.04.019 23602911 PMC3766412

[B53] Wang Y. Li L. Fan L. H. Jing Y. Li J. Ouyang Y. C. (2019). N-Acetyl-L-Cysteine (nac) delays post-ovulatory oocyte aging in mouse. Aging (Albany NY) 11 (7), 2020–2030. 10.18632/aging.101898 30978175 PMC6503888

[B54] Wu L. Wei Y. Li H. Li W. Gu C. Sun J. (2020). The ubiquitination and acetylation of histones are associated with male reproductive disorders induced by chronic exposure to arsenite. Toxicol. Appl. Pharmacol. 408, 115253. 10.1016/j.taap.2020.115253 32991915

[B55] Xiong B. Zhao Y. Beall S. Sadusky A. B. Dean J. (2017). A unique egg cortical granule localization motif is required for ovastacin sequestration to prevent premature Zp2 cleavage and ensure female fertility in mice. PLoS Genet. 13 (1), e1006580. 10.1371/journal.pgen.1006580 28114310 PMC5293279

[B56] Yao S. Lopez-Tello J. Sferruzzi-Perri A. N. (2021). Developmental programming of the female reproductive system-a review. Biol. Reprod., 104(4): 745–770. 10.1093/biolre/ioaa232 33354727

[B57] Yin C. Liu J. He B. Jia L. Gong Y. Guo H. (2019). Heat stress induces distinct responses in porcine cumulus cells and oocytes associated with disrupted gap junction and trans-zonal projection colocalization. J. Cell. Physiol. 234 (4), 4787–4798. 10.1002/jcp.27277 30341896

[B58] Zhang Y. Jiang N. Liu Q. Zhu Y. Huang X. (2020). Role of mitochondrial damage in cadmium-induced cell apoptosis and DNA damage of hepatocytes. Wei Sheng Yan Jiu 49 (2), 290–297. 10.19813/j.cnki.weishengyanjiu.2020.02.021 32290948

[B59] Zhou C. Zhang X. ShiYang X. Wang H. Xiong B. (2019). Tea polyphenol protects against cisplatin-induced meiotic defects in porcine oocytes. Aging (Albany NY) 11 (13), 4706–4719. 10.18632/aging.102084 31301169 PMC6660049

